# Oppositional defiant- and conduct disorder-like problems: neurodevelopmental predictors and genetic background in boys and girls, in a nationwide twin study

**DOI:** 10.7717/peerj.359

**Published:** 2014-04-22

**Authors:** Nóra Kerekes, Sebastian Lundström, Zheng Chang, Armin Tajnia, Patrick Jern, Paul Lichtenstein, Thomas Nilsson, Henrik Anckarsäter

**Affiliations:** 1 CELAM (Centre for Ethics, Law and Mental Health), Institution for Neuroscience and Physiology, University of Gothenburg, Gothenburg, Sweden; 2 Swedish Prison and Probation Service, Research and Development Unit, Gothenburg, Sweden; 3 Gillberg Neuropsychiatric Centre, University of Gothenburg, Gothenburg, Sweden; 4 Department of Medical Epidemiology and Biostatistics, Karolinska Institutet, Stockholm, Sweden; 5 Department of Psychology and Logopedics, Åbo Akademi University, Turku, Finland; 6 Genetic Epidemiology Laboratory, Queensland Institute of Medical Research, Brisbane, Australia

**Keywords:** Oppositional defiant disorder, Conduct disorder, Attention deficit hyperactivity disorder, Autism spectrum disorder, Social interaction, Boys, Girls

## Abstract

**Background.** Previous research has supported gender-specific aetiological factors in oppositional defiant disorder (ODD) and conduct disorder (CD). The aims of this study were to identify gender-specific associations between the behavioural problems–ODD/CD-like problems–and the neurodevelopmental disorders–attention deficit hyperactivity disorder (ADHD), autism spectrum disorder (ASD)–and to investigate underlying genetic effects.

**Methods.** 17,220 twins aged 9 or 12 were screened using the Autism–Tics, AD/HD and other Comorbidities inventory. The main covariates of ODD- and CD-like problems were investigated, and the relative importance of unique versus shared hereditary and environmental effects was estimated using twin model fitting.

**Results.** Social interaction problems (one of the ASD subdomains) was the strongest neurodevelopmental covariate of the behavioural problems in both genders, while ADHD-related hyperactivity/impulsiveness in boys and inattention in girls stood out as important covariates of CD-like problems. Genetic effects accounted for 50%–62% of the variance in behavioural problems, except in CD-like problems in girls (26%). Genetic and environmental effects linked to ADHD and ASD also influenced ODD-like problems in both genders and, to a lesser extent, CD-like problems in boys, but not in girls.

**Conclusions.** The gender-specific patterns should be considered in the assessment and treatment, especially of CD.

## Background

For a diagnosis of oppositional defiant disorder (ODD), a repetitive (persistent) pattern of defiant, disobedient, or hostile behaviour should be observed in a child, while the diagnosis of conduct disorder (CD) entails serious violations of the basic rights of others, social norms, and rules. Prevalence estimates for these behavioural disorders vary widely, because of the broad range of definitions, though reports tend to be consistent in finding an increased prevalence of these disorders in boys (Maughan et al., 2004). This study used a population-based twin cohort to investigate, separately in boys and girls, the relation of ODD and CD with disorders usually referred to as neurodevelopmental, such as attention deficit hyperactivity disorder (ADHD) and autism spectrum disorders (ASD) and to examine any shared aetiologies of these phenotypically different clinical conditions.

There is growing evidence that neurodevelopmental disorders are susceptibility factors or predecessors of oppositional and antisocial aggressive behaviours that begin in childhood. Among these neurodevelopmental disorders, the hyperactive/impulsive subdomain of ADHD shares phenotypic traits (impulsive, under-controlled behaviours) with ODD and CD, and evidence for shared aetiological factors has been reported (Nadder et al., 2002; Tuvblad et al., 2009), even if a strong and unique genetic component has also been discerned behind CD (Lahey et al., 2011). ASD might be associated with ODD and CD through deficient empathy which has been proposed as a key cognitive-emotional deficit in both ASD and the “callous-unemotional” subtype of CD (Frith, 1991). With the help of a detailed description of the overlap between environmental and genetic background factors of neurodevelopmental disorders (ADHD, ASD) on one hand and behavioural problems (ODD, CD) on the other, it will be possible to argue for comprehensive treatment strategies, including pharmacological and/or psychosocial treatment possibilities, in order to alleviate behaviour problems.

While physical aggression is more typical for boys (Campbell, 2006; Lagerspetz, Bjorkqvist & Peltonen, 1988), and relational aggression is more common in girls (Crick & Grotpeter, 1995; Lagerspetz, Bjorkqvist & Peltonen, 1988), the overall symptoms of ODD and CD do not differ between the genders (Maughan et al., 2004). And while the prevalence of ODD and CD is significantly higher in boys than in girls, this difference is reduced in young adulthood, as the early teenage years are a high-risk period for girls to develop aggressive behaviours (Moffitt et al., 2001). In view of the skew between girls and boys, gender-specific diagnostic criteria for ODD and CD have been proposed (Keenan, Coyne & Lahey, 2008). However, because of the limited number of large-scale clinical and epidemiological studies including both genders, the empirical ground for gender-based subdivisions has not yet been proved.

All treatment of mental disorders, in children or in adults, depends on a good understanding of aetiology. It is therefore important to identify the background factors of ODD and CD, including specific patterns of genetic and environmental susceptibilities.

This study aimed to investigate separately in boys and girls:

1. the prevalence of ODD/CD and the age at onset of these problems;
2. the association between ODD/CD and the two subdomains of ADHD (concentration/attention and activity/impulsiveness) and the three subdomains of ASD (social interaction, flexibility, and language); and
3. the role of genetic and environmental aetiologies in ADHD or ASD in common with those of ODD/CD.

## Methods

### Subjects

This paper is based on data from the ongoing Child and Adolescent Twin Study in Sweden (CATSS), a longitudinal, nationwide twin study on somatic and mental health problems in childhood (Anckarsäter et al., 2011). Since July 2004, parents of all Swedish-born twins have been asked on the occasion of the twin’s 9th birthday to participate in a telephone interview on their somatic and mental health and their psychosocial and family circumstances. During the first three years of the study 12-year-old twins were also included. Data for this paper was retrieved in January 2010, when 8,610 informants responded for 17,220 individual twins, corresponding to 80% of the eligible individuals. In the analyses presented here, 156 children with early brain damage syndromes and 22 with well-known chromosomal aberrations were excluded, resulting in a final study population of 17,042 children with a close to equal gender distribution and a 3:2 ratio of those aged 9 and 12.

Zygosity was determined by a validated algorithm with a >95% predictive value compared to DNA-testing (Hannelius et al., 2007). After the telephone interview two saliva collecting kits were sent to the participating families, where twins could send in their saliva sample if they wished. Only twins with more than 95% probability of being correctly classified were assigned a zygosity by this method, while those with uncertain zygosity were retained in a separate group. Of the study population, 26.8% were monozygotic (MZ), 34.9% were same-sex dizygotic (DZ), 34% were opposite-sex DZ, and 4.3% were of an unknown zygosity.

### Measures

#### Autism–Tics, AD/HD and other comorbidities inventory

In the CATSS telephone interviews, parents responded to the Autism–Tics, AD/HD and other Comorbidities inventory (A-TAC). The A-TAC includes 96 questions that cover a broad range of childhood psychiatric problems organized into modules corresponding to DSM-IV diagnoses usually identified in infancy and early childhood. Questions are worded to reflect DSM-IV criteria and clinical features, and are answered from a lifetime perspective with choices (and corresponding points) that range from “no” (0) through “yes, to some extent” (0.5), to “yes” (1.0). By adding the scores for each module, the A-TAC provides continuous measures for each condition. When a parent answered “yes, to some extent” or “yes” to any question in a module, several follow-up questions were asked, including, “When did you first notice the problems we just asked about?”

The ODD and CD scales of the A-TAC consist of five items each, both with good to acceptable internal consistency (Cronbach’s alpha = 0.75 (Hansson et al., 2005)). These two scales reflect DSM-IV criteria for the proxy diagnoses of ODD and CD, but were not included in reported analyses of previous validation studies of clinical diagnoses, because the prevalence of these conditions was too low in the groups studied. Cut-offs for the determination of the prevalence of ODD- and CD-like problems were established with the help of analyses of the A-TAC data in a clinical population of adolescents, enriched for these behavioural problems, in relation to the control group from the concomitantly collected validation study (Larson et al., 2010). The A-TAC data was collected from a group of institutionalized adolescents (*n* = 66) when there was an informant who could verify the appropriate information (i.e., had known the adolescent as a child) (Stahlberg, Anckarsater & Nilsson, 2010). All subjects in this group were considered to meet the DSM-IV criteria for ODD, as all had met the legal prerequisites for institutionalization (e.g., rule-breaking, uncooperativeness, disruptiveness, truancy). CD diagnoses were assigned to individuals with more than three documented types of norm-breaking behaviour and/or criminality. The telephone interview version of A-TAC was often difficult to carry out, as the children were known to have a number of problems, therefore informants (parents or caregivers) were either interviewed in person by a psychologist or asked to fill out the written A-TAC questionnaire with the help of a psychologist or institution staff member if necessary. Both the ODD and CD scales showed excellent overall predictive ability in receiver operating characteristics (ROC) analyses (area under the curve [AUC] = 0.89 for ODD and 0.95 for CD), indicating construct validity. To convert the ODD score to a dichotomous category (to be used here as a research proxy for clinical diagnosis and referred to as “ODD-like problems”), a cut-off of ≥ 3 was selected yielding a sensitivity of 0.51 and a specificity of 0.96. For the corresponding research proxy of CD (referred to as CD-like problems), a cut-off of ≥ 2 was identified, yielding a sensitivity of 0.55 and a specificity of 0.98. As the identified cut-offs of both of these scales had high specificities, there is a high probability that twins identified by these cut-offs had behavioural problems corresponding to DSM-IV diagnostic criteria for ODD and CD.

The ADHD scale of the A-TAC contains two modules, one with nine items corresponding to the concentration/attention subdomain, and one with ten items corresponding to the activity/impulsiveness subdomain. The ASD scale of the A-TAC contains three modules: the language impairments and social interaction problems modules with six items each, and the flexibility problems module with five items. The psychometric properties of these scales (ADHD and ASD) have been reported in previous studies, showing good to excellent internal and external validity (Anckarsäter et al., 2011; Larson et al., 2010).

### Data analysis

All statistical analyses were performed using SPSS 19.0 (IBM) and Mx (Neal et al., 2003).

Descriptive statistics were calculated for the whole study population and for boys and girls separately.

To investigate the effects of ADHD and ASD on ODD- and CD-like problems, univariable and multivariable regressions were performed using generalized estimating equations to control for dependence within twins. As the dependent variables the dichotomous variables (ODD- and CD-like problems) were used and binary response models were fitted to the data. All continuous predictor variables were inserted in the model as covariates, while the ordinal variable “age” was inserted as a cofactor (i.e., aged 9 or 12), and their main effects were analysed in both univariable and multivariable models. The univariable model calculates the risk for the outcome (ODD-like problems or CD-like problems) when only one predictor (neurodevelopmental problem) is present in a child. The multivariable model on the other hand calculates the risk for the outcome, when all predictors are considered. Odds ratios (OR) could be interpreted as risk ratios because the prevalence if the dependent variables (ODD- or CD-like problems) were small in the study population.

Twin modelling is based on the variance and covariance in twins by comparing MZ twins and DZ twins. Typically, twin modelling is used to decompose the variance of each phenotype, as well as the covariance between phenotypes, into additive genetic factors (A), dominant genetic factors (D), shared environmental factors (C), and non-shared environmental factors (E) (Neal et al., 2003).

To disentangle the genetic and environmental influences on, ODD-like problems, CD-like problems and the neurodevelopmental disorders, intra-class correlations were calculated and univariate ACE-models were fitted on dimensional scores (Table 3). In a second step, cross-twin, cross-trait correlations and phenotypic correlations were calculated (Table 3), and we used a bivariate Cholesky decomposition to estimate the extent of the common genetic and environmental influences between ODD-like problems, CD-like problems, and the neurodevelopmental disorders. Prior to model fitting, subscales were corrected for the effect of interview order using a regression, and to account for the skewed distribution of the examined traits, data was logarithmically transformed and a contrast model taking sibling interaction into consideration was included. As most of the twin correlations suggested that the C component was inconspicuous, AE models were chosen for all bivariate models, with the exception of CD-like problems in girls, where the C component was prominent. We did not attempt to test reduced models, since this can lead to bias and inaccuracy in the observed estimates (Sullivan & Eaves, 2002). A root-mean squared error of <0.05 generally indicates a good fit and is appropriate to use in large data samples (Mulaik et al., 1989).

### Ethical considerations

The study was designed in accordance with the Helsinki declaration and approved by the ethical review board of Karolinska Institutet (Dnr: 02-289). All participants (parents or guardians of children) consented to the study after receiving written and oral information. All analyses were performed using anonymized data files.

## Results

### Prevalence, age at onset, and overlap

A total of 476 children (3% of the study population) scored above the cut-off for ODD-like problems and 159 (1%) above that for CD-like problems. The prevalence of both ODD-like problems and CD-like problems was higher in boys than in girls (3.5% compared to 2.1% for ODD-like problems, OR = 1.66; CI = 1.37–2.01, and 1.3% compared to 0.6% for CD-like problems, OR = 2.10; CI = 1.47–2.99, respectively). Sixty-three per cent of those who scored above the cut-off for ODD-like problems (*n* = 301) and 68% of those who scored above the cut-off for CD-like problems (*n* = 109) were boys. Both problems coexisting were found in 67 of the boys and 28 of the girls.

**Figure 1:**
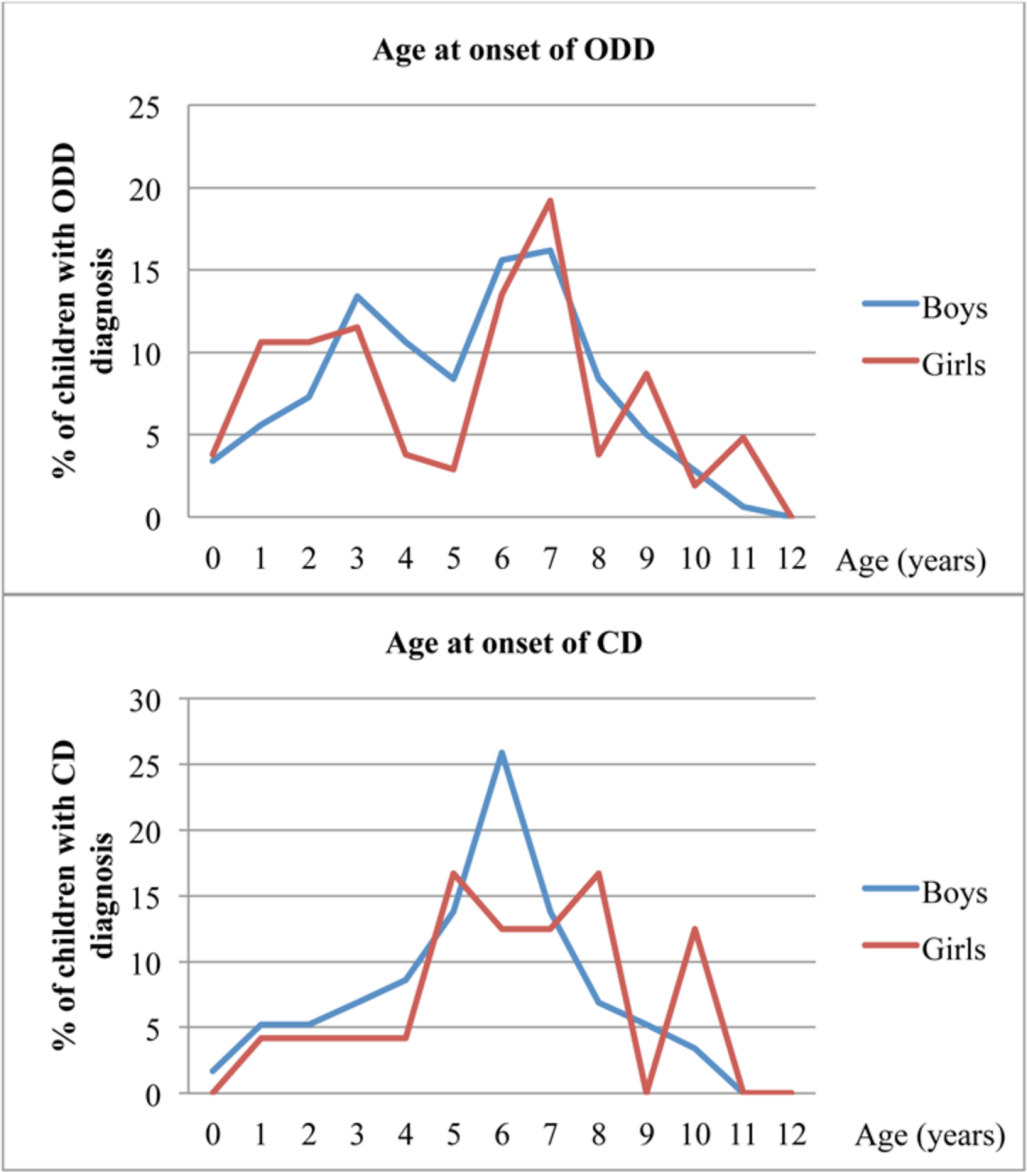
Age at onset of ODD- and CD-like problems in the CATSS, as reported by parents. Notes: ODD, oppositional defiant disorder-like problems; CD, conduct disorder-like problems; CATSS, child and adolescent twin study in sweden.

There were two peaks for the onset of ODD-like problems, around age 3 (between ages 1 and 3 in girls) and then between ages 6 and 7 in both genders (Fig. 1). The peak age of onset for CD-like problems was 6 years of age in boys, with onset in 15 of the 58 boys who reached cut-off for CD-like problems (missing data for 51 boys); the 24 girls who reached cut-off for CD-like problems had a fairly even but inconclusive pattern of age of onset (missing data for 26 girls) (Fig. 1).

### Neurodevelopmental problems associated with ODD- and CD-like problems

In univariable models, all scores of neurodevelopmental problems (A-TAC module scores of ADHD and ASD) were significantly positively associated with ODD-like problems and CD-like problems, in both genders (Tables 1 and 2). For example, for each new concentration/attention symptom, the risk for ODD-like problems increased by 66% in boys and by 85% in girls (OR = 1.66/1.85; CI = 1.59–1.73/1.75–1.95, respectively), while each and every new point in the social interaction scale increased the risk for the presence of CD-like problems in boys with 206% (OR = 3.06; CI = 2.74–3.42) and in girls with 252% (OR = 3.52; CI = 2.91–4.25).

In a multivariable model for ODD-like problems, all module scores (except the ASD language problems module, which was not a risk factor in girls) retained their significant positive associations in both genders. For the presence of CD-like problems, the multivariable model identified the ASD social interaction module as the strongest risk factor in boys, with each new symptom increasing the risk by 109% (OR = 2.09; CI = 1.63–2.67). The ADHD activity/impulsiveness module was a somewhat weaker, but still significant, risk factor for boys (Table 1). For girls, the ADHD concentration/attention module was the strongest risk factor, and for each new symptom in this module the risk for CD-like problems increased by 66% (OR = 1.66; CI = 1.38–1.99). A weak but significant association with the ASD module social interaction was also found in the multivariable model for girls (Table 2).

**Table 1:** Associations, indicated as odds ratios, between the dependent variable ODD- or CD-like problems and the independent variables of ADHD and ASD modules. General estimated equation models for boys.

Variable	Crude measures				Univariable model		Multivariable model^a^		Univariable model		Multivariable model^a^	
	N	Min/Max	M	SD	OR	95% CI	OR	95% CI	OR	95% CI	OR	95% CI
	**BOYS**				**ODD**				**CD**			
Concentration/attention	8653	0–9	1.25	1.90	1.66^***^	1.59–1.73	1.16^***^	1.07–1.25	1.68^***^	1.58–1.79	1.10	0.98–1.24
Activity/impulsiveness	8654	0–10	1.10	1.79	1.72^***^	1.65–1.80	1.32^***^	1.23–1.41	1.78^***^	1.68–1.89	1.45^***^	1.32–1.59
Social interaction problems	8648	0–6	0.31	0.68	3.47^***^	3.08–3.92	1.92^***^	1.55–2.38	3.06^***^	2.74–3.42	2.09^***^	1.63–2.67
Flexibility problems	8659	0–5	0.31	0.68	3.39^***^	3.01–3.82	1.68^***^	1.40–2.04	2.62^***^	2.31–2.98	1.02	0.79–1.31
Language problems	8656	0–6	0.30	0.66	2.42^***^	2.17–2.69	0.70^**^	0.56–0.88	2.25^***^	2.00–2.53	0.78^*^	0.62–0.99

**Notes.**

ODD oppositional defiant disorder-like problems CD conduct disorder-like problems OR odds ratio CI confidence interval

a Adjusted for age.

* *P* < 0.05.

** *P* < 0.01.

*** *P* < 0.001.

**Table 2:** Associations, indicated as odds ratios, between the dependent variable ODD- or CD-like problems and the independent variables of ADHD and ASD modules. General estimated equation models for girls.

Variable	Crude measures				Univariable model		Multivariable model ^a^		Univariable model		Multivariable model ^a^	
	N	Min/Max	M	SD	OR	95% CI	OR	95% CI	OR	95% CI	OR	95% CI
	**GIRLS**				**ODD**				**CD**			
Concentration/attention	8318	0–9	0.74	1.44	1.85^***^	1.75–1.95	1.22^***^	1.09–1.36	2.03^***^	1.86–2.23	1.66^***^	1.38–1.99
Activity/impulsiveness	8314	0–10	0.71	1.40	1.86^***^	1.76–1.97	1.35^***^	1.23–1.48	1.79^***^	1.65–1.93	1.17	1.00–1.36
Social interaction problems	8305	0–6	0.21	0.51	5.01^***^	4.16–6.05	2.36^***^	1.77–3.14	3.52^***^	2.91–4.25	1.61^*^	1.10–2.35
Flexibility problems	8321	0–5	0.16	0.46	4.54^***^	3.77–5.46	1.85^***^	1.39–2.45	3.03^***^	2.48–3.71	0.96	0.64–1.45
Language problems	8321	0–6	0.19	0.47	3.32^***^	2.79–3.95	0.79	0.57–1.10	2.88^***^	2.36–3.52	0.86	0.55–1.33

**Notes.**

ODD oppositional defiant disorder-like problems CD conduct disorder-like problems OR odds ratio CI confidence interval

a Adjusted for age.

* *P* < 0.05.

** *P* < 0.01.

*** *P* < 0.001.

### Genetic and environmental factors in ODD, CD, ADHD and ASD

Univariate ACE models were calculated on the dimensional A-TAC scores for ODD- and CD-like problems and the two neurodevelopmental-problem areas (ADHD and ASD) (Table 3). Generally, a strong genetic component was found in all of these scores, accounting for about 60% to 70% of the variance in boys and around 50% to 60% in girls, with the notable exception of CD-like problems in girls, in whom the genetic component accounted for only 26% of the variance, the shared environmental factor accounted for 25%, and the non-shared environmental factor accounted for 48% of variance. No common environmental factors were detected for any of the other conditions.

**Table 3:** Twin correlations and heritability estimates, analysed separately for boys and girls.

Boys					Girls			
**Intra-class correlations (95% CI)**								
	**ODD**	**CD**	**ADHD**	**ASD**	**ODD**	**CD**	**ADHD**	**ASD**
MZ	.59(.56–.64)	.62(.58–.65)	.68(.65–.71)	.72(.69–.75)	.47(.43–.52)	.44(.40–.49)	.58(.54–.62)	.55(.51–.59)
DZ	.29(.24–.33)	.32(.28–.37)	.19(.14–.23)	.24(.19–.29)	.24(.19–.29)	.43(.39–.47)	.19(.14–.24)	.32(.27–.37)
**Univariate analyses (95% CI:s)**								
A	.61(.55–.64)	.67(.63–.70)	.67(.63–.70)	.72(.69–.75)	.50(.42–.54)	.26(.15–.38)	.61(.58–.65)	.59(.53–.63)
C	.00(.00–.05)	.00(.00–.03)	.00(.00–.03)	.00(.00–.01)	.00(.00–.05)	.25(.17–.34)	.00(.00–.01)	.00(.00–.04)
E	.39(.36–.42)	.33(.30–.36)	.33(.30–.37)	.28(.25–.31)	.50(.46–.54)	.48(.44–.53)	.39(.35–.42)	.41(.37–.41)
**Cross-twin cross-trait correlations (95% CI:s)**								
	**ODD/ADHD**	**ODD/ASD**	**CD/ADHD**	**CD/ASD**	**ODD/ADHD**	**ODD/ASD**	**CD/ADHD**	**CD/ASD**
MZ	.47(.42–.51)	.45(.41–.50)	.30(.24–.35)	.32(.27–.39)	.38(.33–.42)	.38(.32–.42)	.22(.16–.27)	.21(.16–.26)
DZ	.23(.19–.28)	.23(.18–.28)	.18(.13–.22)	.24(.19–.28)	.23(.18–.28)	.26(.21–.31)	.18(.13–.23)	.18(.13-24)
**Phenotypic correlations (95% CI:s)**								
	.60(.58–.62)	.62(.60–.65)	.44(.41–.47)	.48(.45–.51)	.52(.50–.55)	.56(.53–.59)	.44(.41–.47)	.38(.35–.41)

**Notes.**

A additive genetic effects C shared environmental effects E non-shared environmental effects CI confidence interval ODD oppositional defiant disorder-like problems CD conduct disorder-like problems ADHD attention deficit hyperactivity disorder ASD autism spectrum disorder

**Figure 2:**
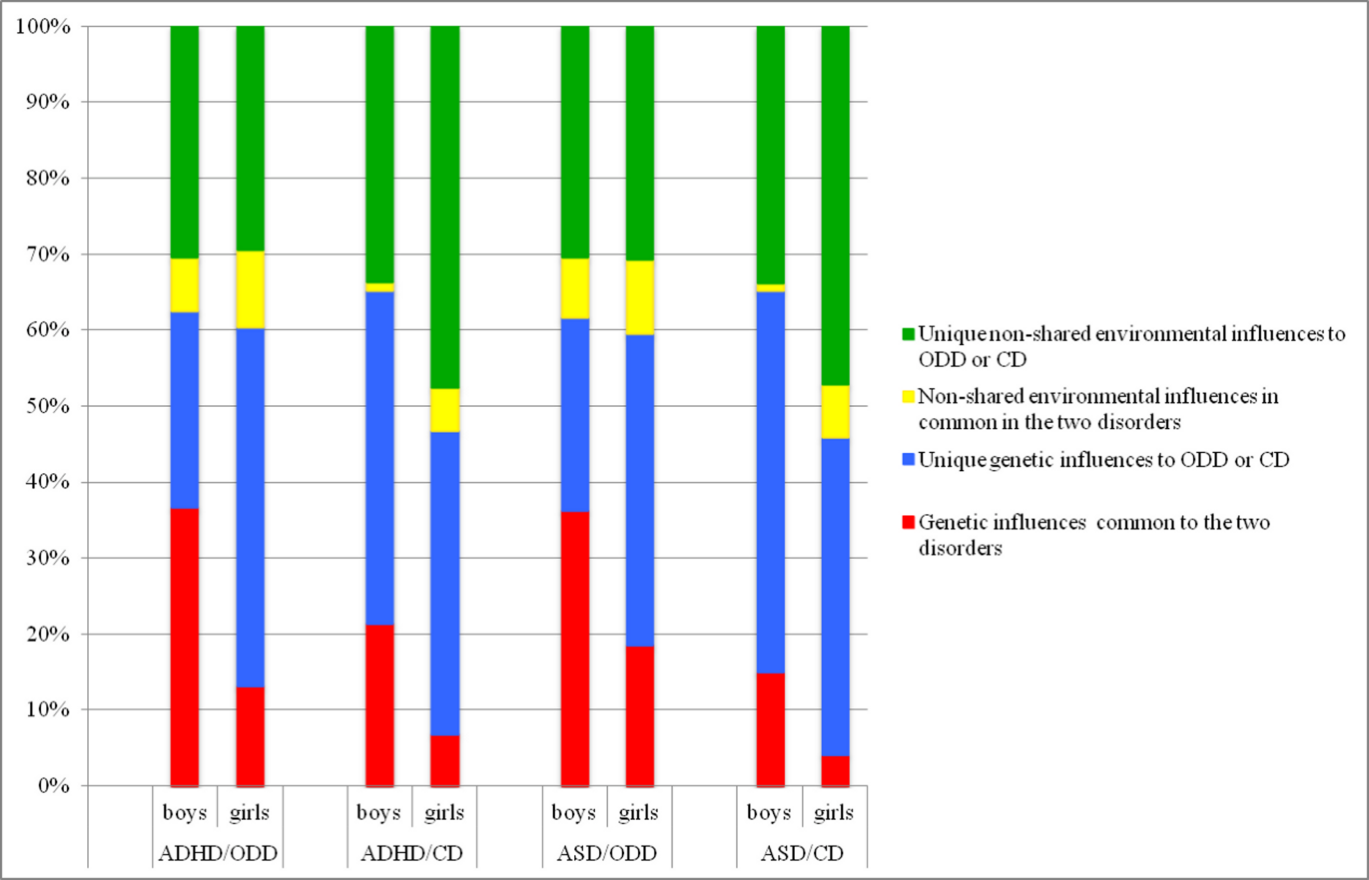
Variance in liability between ODD- or CD problems and ADHD, and between ODD- or CD-problems and ASD, analyzed separately in boys and girls. Notes: ODD, oppositional defiant disorder-like problems; CD, conduct disorder-like problems; ADHD, attention deficit hyperactivity disorder; ASD, autism spectrum disorder

The overlap of genetic and environmental factors influencing ODD- or CD-like problems and the neurodevelopmental problems captured by ADHD or ASD scores were quantified by gender as shown in Fig. 2. The cross-twin, cross-trait correlations were larger for MZ than for DZ twins for all traits except CD/ADHD (MZ 0.22, CI [0.16–0.27]; DZ 0.18, CI [0.13–0.23]) and CD/ASD (MZ 0.21, CI [0.16–0.26]; DZ 0.18, CI [0.13–0.24]) in girls, in whom the estimates were fairly similar. The phenotypic correlation was higher between ADHD/ASD and ODD-like problems (0.52–0.62) than ADHD/ASD and CD-like problems (0.38–0.48) in both boys and girls. In all analyses the majority of explained variance was due to unique non-shared genetic and environmental effects (Fig. 2). In boys, 37% of the variance in phenotypic correlations could be attributed to genetic effects common to both ADHD and ODD-like problems, and 21% to those common to ADHD and CD-like problems. The corresponding figures for girls were 13% and 7%. Moreover, 36% of the variance in phenotypic correlations could be attributed to genetic effects common to both ASD and ODD-like problems, and 15% to those common to ADHD and CD-like problems, in boys, while the corresponding figures for girls were 19% and 4%.

## Discussion

### Prevalence and age at onset of ODD-like problems and CD-like problems in boys and girls

This study analysed the prevalence, age at onset, neuropsychiatric predictors, and aetiology of ODD- and CD-like problems, with a focus on gender-specificity, in a 12-year-old (40%) and 9-year-old (60%) twin population. The prevalence of ODD-like problems in boys (3.5%) was very similar to previous findings (for review see Maughan et al., 2004). However, in our study the prevalence of ODD-like problems (2.1%) and CD-like problems (0.6%) in girls and for CD-like problems in boys (1.3%) was lower than had previously been found in children aged 8 to 14 years (for review see Maughan et al., 2004). The generally lower presentation of behavioural problems in our study population may be explained by the source of our data (parental reports) and could suggest that parents might not be capable of an accurate appraisal of their children’s behaviour outside of the family context, e.g., at school or among their peers (Breton et al., 1999). Another possible explanation is that our study population was more representative of the prevalence of these problems in younger ages, because most of our study population were 9-year-olds. This explanation is also supported by a study on the prevalence of ODD and CD in children aged 9 to 11 years, in which the prevalence of these conditions, based on parental reports, showed figures in the lower end, very similar to our results, especially for CD (Breton et al., 1999).

The young age of our population explains why we were unable to capture the two well-known early and late ages of onsets for CD (DSM-IV); although the early onset of CD-like problems could be captured in this young population, the late onset age of CD-like problems was underrepresented due to the lower proportion of adolescent subjects. For somewhat more than 25% of the boys with CD-like problems, the onset of problems was reported to have occurred at 6 years of age. For girls we could not define any peak of age at onset for CD-like problems, which probably is due to a relatively small frequency of CD-like problems in girls. If the study population had consisted of older subjects (aged from 12 to 15) we probably would have found a peak for girls as well, as most girls with aggressive behaviours tend to develop their problems as teenagers (Moffitt et al., 2001). For ODD-like problems we were able to measure two peaks of age at onset, an early start at age 2 to 3, related to oppositional acts, and a later peak at age 6 to 7 that might represent a more emotion-driven aggressive opposition.

### Neurodevelopmental problems associated with child aggressive behaviours and gender aspects

Autistic-like social interaction problems were implicated as among the strongest neurodevelopmental covariates of ODD- and CD-like problems in both genders, while ADHD-related hyperactivity/impulsiveness in boys and inattention in girls stood out as important covariates of CD-like problems. Prior research has implicated autistic-like traits and ASD in juvenile delinquency (Geluk et al., 2012) and in CD (Lundstrom et al., 2011). A way of testing whether social interaction problems in children with ODD/CD-like problems are autistic behaviours or merely a consequence of ADHD would be to study these problems in subjects who receive treatment for ADHD. Successful treatment of ADHD would lead to both a reduction of social interaction problems due to hyperactivity/impulsiveness and a better recognition of the role of primary deficits in social cognition when the effect of poor attention is neutralized. However, supporting the notion that social interaction problems really are related to the autism spectrum, there is widespread clinical experience that pharmacological treatment of ADHD may “unravel” or aggravate, rather than reduce, autistic-like traits (Grzadzinski et al., 2011; Mayes et al., 2012). Specific systematic studies are required before any conclusive statements may be made in that regard.

Interestingly, ASD-related language problems in the presence of ADHD tend to carry a decreased risk for ODD- and CD-like problems, especially in boys. This protective effect could be explained by the possible decreased social interaction that is a consequence of language problems, or by explicit guidance and support from adults (e.g., preschool teachers) in response to these obvious problems. As a result of either of these conditions, situations and interactions that would otherwise have led to frustration and aggressive reactions are minimized and thus the breeding ground for aggressive behaviours may be eliminated.

The stronger genetic effects and aetiological overlaps across neurodevelopmental disorders and ODD/CD-like problems in boys raise questions about possible gender-specific risk/protective factors. For boys the strongest risk factor/predictor of CD-like problems was the cluster of autistic-like problems in social interaction, while for girls it was the facet describing concentration/attention problems. (It should be noted that concentration/attention problem was not a significant predictor for CD-like problems in boys in the multivariable model.) Activity/impulsiveness has been proposed as a more salient feature of ADHD in boys than in girls, presumable because girls may have protective neurobiological mechanisms or be subjected to stronger social pressures that force them to counteract restlessness and impulsiveness. The gender difference in the role of inattention for CD-like problems may partly be confounded by previously hypothesized, potentially gender-related, biased expectations of parents and teachers, according to which they might have stricter or higher expectations of girls and therefore tend to exaggerate smaller behavioural deviations by enlarged scores.

### Gender-specificity of the etiological factors

With the exception of CD-like problems in girls, the genetic components in both genders were almost as strong for ODD/CD-like problems as for neurodevelopmental problems, which is in line with a number of previous twin studies (Lahey et al., 2011; Meier et al., 2011; Tuvblad et al., 2009). Genetic effects seem to be more pronounced in boys and, according to the multiple threshold theory, boys have a lower threshold for the expression of neurodevelopmental and conduct disorders, which may explain the higher prevalence of these disorders in boys than in girls (Rhee et al., 1999). It has also been suggested that the specific risk factors may differ (Meier et al., 2011). CD-like problems in girls differed from CD-like problems in boys both at the phenotypic level and in the aetiology. In girls, CD-like problems were moderately affected by both genetic factors and shared environmental influences, which were not found to affect any other condition in the study. A specific aetiology in girls, with a substantial contribution of shared environmental factors, was previously found for the callous-unemotional dimension of CD (Fontaine et al., 2010). Other studies, however, have found that very similar proportions of genetic and environmental influences explain the variance of CD in both genders, even though the specific risk factors may differ for boys and girls (Meier et al., 2011). This is supported in the bivariate analyses, in which the association between CD-like problems and ASD or ADHD showed small common genetic effects and large unique environmental effects. For boys the etiological relationships between ASD and ODD-like problems and ADHD and ODD-like problems were quite similar, as were those for CD-like problems and ASD or ADHD (albeit smaller). Molecular genetic studies examining ODD or CD should also investigate ASD and ADHD.

### Strengths and limitations

A number of strengths of this study deserve to be emphasized: the large sample size; the genetically sensitive design; and the high (80%) response rate, which makes our generalizations more reliable.

Several limitations should also be mentioned. First, the findings are based on retrospective parental reports. Although the A-TAC inventory is well validated for ADHD and ASD, a clinical diagnostic interview would nevertheless be preferable for ODD and CD as well; however, this is not feasible in large-scale population studies. Second, even if twin populations do not differ in many respects from the general population, recent investigations point to the possibility of twins being at a lower risk for starting substance abuse or criminality than age-matched singletons (Hjern et al., 2012). This suggests that our results might slightly underestimate the prevalence of ODD- and CD-like problems in the general population for two reasons: (1) using cut-offs coupled with very high specificity, but reasonably low sensitivity, and (2) using twin populations that probably have a slightly lower prevalence of disruptive problems.

## Conclusions

### Clinical implications

First, gender-specific patterns should be considered in the assessment and treatment of ODD and especially in CD, which showed the largest differences between boys and girls. Since inattention, which is a less disruptive behaviour pattern than activity/impulsiveness but still a risk factor for CD in girls, is a discreet problem and easy to overlook, there is a risk that girls who may later develop conduct problems might go unnoticed. Moreover, it has been shown that the disruptive behaviour of boys tends to occur independently of the social context; boys with disruptive behaviours display these in every type of social environment. Girls’ disruptive behaviours, however, are more dependent on the social context; girls seem more able than boys to adapt to the expectations of an “outsider/examiner”, while in interaction with their mothers they display even more disruptive behaviours than boys (Gray et al., 2012). These results together with our results highlight the importance of a gender-specific and social context-dependent conceptualization of disruptive behaviours.

Second, in children with ODD and/or CD, clinicians should systematically assess for neurodevelopmental problems, with a specific focus on social interaction problems. Clinical assessment of children and adolescents affected by ODD and/or CD should therefore cover a broad array of behavioural and neurocognitive problems and aim to identify all co-existing types of neurodevelopmental problems. ADHD is the most well known coexisting disorder, and has an established pharmacological treatment strategy, but it is equally important to recognize autistic-like traits or ASD, as children with these conditions may be helped by a structured environment, psycho-education, and better understanding of their different cognitive strategies. It seems especially important that existing behavioural family intervention programmes (e.g., the Webster-Stratton group programme or the telephone-assisted Sanders’ interventions) aimed to improve social interaction and communication abilities, and multimodal treatment strategies, including the combination of central stimulant treatment and behaviour therapy for children with ADHD (Jensen et al., 1999), are offered as early as possible.
